# Can the Consumption of Ultra-Processed Food Be Associated with Anthropometric Indicators of Obesity and Blood Pressure in Children 7 to 10 Years Old?

**DOI:** 10.3390/foods9111567

**Published:** 2020-10-28

**Authors:** Tafnes Oliveira, Isabella Ribeiro, Gabriela Jurema-Santos, Isabele Nobre, Ravi Santos, Camilla Rodrigues, Kevin Oliveira, Rafael Henrique, Wylla Ferreira-e-Silva, Alice Araújo

**Affiliations:** 1 Department of Nutrition, Universidade Federal de Pernambuco (UFPE), Recife-PE 50670-901, Brazil; tafnes.lais@ufpe.br (T.O.); isabella.ribeiro@ufpe.br (I.R.); gabriela.cjsantos@ufpe.br (G.J.-S.); belegn@hotmail.com (I.N.); ravi.santos@ufpe.br (R.S.); 2 Department of Nutrition, Centro Acadêmico de Vitória (CAV)-Universidade Federal de Pernambuco (UFPE), Vitória de Santo Antão-PE 55608-680, Brazil; camilla.peixoto@ufpe.br (C.R.); kevin.oliveira@ufpe.br (K.O.); wylla.silva@ufpe.br (W.F.S.); 3 Department of Physical Education, Universidade Federal de Pernambuco (UFPE), Recife-PE 50670-901, Brazil; rafael.shenrique@ufpe.br; 4 Department of Public Health, Centro Acadêmico de Vitória (CAV)-Universidade Federal de Pernambuco (UFPE), Vitória de Santo Antão-PE 55608-680, Brazil

**Keywords:** food processing, food consumption, schoolchildren, overweight, hypertension

## Abstract

The consumption of ultra-processed foods plays an important role in the development of obesity and hypertension. The present study investigated the association between consumption of food according to the degree of processing and anthropometric indicators of obesity and blood pressure in children. This is a cross-sectional study with 164 children aged 7–10 years. The body mass index (BMI) for age, waist circumference (WC), and waist-to-height ratio (WHtR) was evaluated. Food consumption was analyzed by three 24-h dietary recalls, and classified as: G1—unprocessed or minimally processed; G2—culinary ingredients and processed food; and G3—ultra-processed food. Linear regression analyses were used to investigate the associations among variables. The average energy consumption was 1762.76 kcal/day, split into 45.42%, 10.88%, and 43.70%, provided by G1, G2, and G3, respectively. Adjusted linear regression analyses identified that the caloric contribution of G1 was inversely associated with DBP, showing that for each 10% increase in the energy intake of minimally processed foods, there was a reduction of 0.96 mmHg in the DBP (β:−0.10; 95% CI:−0.19 to −0.01; r^2^ = 0.20). There was no association between the caloric contribution of food groups and BMI, WC, WHtR, and SBP. Increasing consumption of G1 could be a strategy for the prevention and treatment of hypertension in schoolchildren.

## 1. Introduction

Obesity [1] and systemic arterial hypertension (SAH) [2] are public health problems with an increasing prevalence in children and adolescents worldwide. Between 1975 and 2016, obesity in children and adolescents (5–19 years) increased more than tenfold, from 11 million to around 124 million in the world [1]. In Brazil, it was estimated that, in 2016, the prevalence of obesity in girls and boys (5–9 years) was 12.4% and 17.6%, respectively [3]. The worldwide prevalence of SAH in children and adolescents was 4% according to a meta-analysis performed by Song et al. [2], who also observed a trend of increasing prevalence over the past two decades [2]. National estimates for SAH range from 2.1% to 8.6% in schoolchildren [4,5,6]. In addition, it is known that obesity and high blood pressure in childhood can persist into adolescence [7] and adulthood [8,9,10].

Eating habits play an important role in the development of obesity and SAH in children [6,11]. Among dietary factors, the consumption of ultra-processed foods has received attention [12], mainly due to its low nutritional quality. An augmented contribution of this type of food was positively associated with energetic density, added sugar, sodium, and total and trans fats, as well as being negatively associated with protein and fiber consumption [13,14]. Ultra-processed foods are industrial formulations made with multiple processing steps including some ingredients that make the food more palatable, durable, and cheaper [15]. Studies have shown that the consumption of this type of food is increasing worldwide [16,17,18]. A study with Brazilian children (up to 10 years old) showed that almost half of the calories they ingested (47%) were provided by ultra-processed foods [19].

Investigations about the impacts of food processing on human health are necessary. In adults, a positive association has been documented between the consumption of ultra-processed food and the risk of weight gain [20], overweight/obesity [21], and hypertension [22]. In adolescents, the high consumption of ultra-processed foods was associated with a higher prevalence of metabolic syndrome [23], while the ingestion of minimally processed foods was inversely associated with overweight [24]. Although longitudinal studies with Brazilian children associated the consumption of ultra-processed foods with increased total cholesterol and low-density lipoprotein (LDL) [25], triglycerides [26], and waist circumference [27], the influence of different degrees of food processing on indicators of obesity and blood pressure in children is still poorly described.

Few studies have evaluated the effects of specific types of ultra-processed food on the health of children and adolescents. It was observed that an increase of one serving/day of sugar-sweetened beverages was associated with an increase of 0.8 mmHg in systolic blood pressure (95% CI: 0.4–1.2) and 0.3 (95% CI: 0.0–0.5) mmHg in diastolic blood pressure [28]. Pérez-Gimeno et al. [29] observed that diastolic hypertension was associated with a higher frequency of consumption of salty foods (700 mg of sodium/100 g), such as pizza, chips, and sausages, in Spanish children aged 5–16 years, independent of nutritional status.

We hypothesized that the consumption of ultra-processed food is positively associated with anthropometric indicators of obesity and systolic and diastolic blood pressure in school-age children. Therefore, the aim of this study was to investigate the association between food consumption according to the degree of processing and anthropometric indicators of obesity and blood pressure in children aged 7–10 years.

## 2. Materials and Methods

### 2.1. Sample and Study Design

This is a cross-sectional study, performed from September 2018 to November 2019, which evaluated 164 children (67 boys and 97 girls), aged between 7 and 10 years of age (mean = 8.64 ± 0.88), from five public schools in Vitória de Santo Antão, Pernambuco, Brazil. This study is part of the research project entitled Grow up with Health in Vitória de Santo Antão, Pernambuco, developed since 2009 in the city, with a focus on growth and development, nutritional status, food consumption, biochemical profile, motor development, physical fitness, and the effects of plyometric physical training on these variables in schoolchildren. The municipality has approximately 129,974 inhabitants [30] and the Municipal Human Development Index (MHDI) was 0.640 in 2010, which is considered average [31].

The sample was selected using a non-probabilistic sampling process. In this study, the sample’s statistical power was performed a posteriori using the software GPower 3.1.9 (http://www.gpower.hhu.de/). All the analyses performed showed satisfactory power (>80%), 86–89 considering the sample of 164 and 7 predictors. Schoolchildren aged 7–10 years and regularly enrolled in the municipal public schools were included in the study. Children were excluded if they presented: psychological or behavioral disturbances, physical inability to perform the anthropometric measures, used any medication or presented any disease that could compromise food ingestion, nutritional status, and/or blood pressure, and girls that presented premature menarche. The following were considered as study losses: children who were transferred from the school before all the measures had been performed; children who were not found in four attempts; who had difficulty in answering the 24-h dietary recalls (R24h); who presented incomplete or discrepant data in the evaluation form; and children who refused to continue the study.

This study was approved by the 98–100 Committee from the Federal University of Pernambuco (number 3297655) and was carried out according to the declaration of Helsinki. Both parents and children signed a written informed consent form.

### 2.2. Anthropometric Indicators of Obesity

Body mass and height were measured according to a previous protocol [32], using a digital scale (precision: 100 g; Omron^®^, HBF-214LA, São Paulo, Brazil) and a portable stadiometer (precision: 0.1 cm; MD^®^, HT-01, São Paulo, Brazil). These measures were used to identify the body mass index [BMI = body mass (kg)/height (m^2^)]. Subsequently, the standardized BMI scores were calculated using AnthroPlus software (World Health Organization, Geneva, Switzerland, version 1.0.3). Children were classified into groups: thinness (z-score < −2), eutrophic (≥−2 z-score ≤ +1), overweight (>+1 z-score ≤ +2), and obesity (z-score > +2), according to age and sex [33].

Waist circumference was measured using a measuring tape (scale: 0–200 cm; precision: 0.1 mm; Cescorf^®^, Porto Alegre, Brazil) placed on the midpoint between the lower rib and the superior border of the iliac crest [34]. The measurements were performed twice by the same evaluator and the mean value was calculated. In case of a difference greater than 2.0 cm between the measures, a third one was performed and the two closest measures were considered to calculate the mean. Measurements greater than or equal to the 90th percentile for sex and age were considered abdominal obesity [35]. The waist-to-height ratio (WHtR) was calculated by dividing the waist circumference (cm) by the height (cm). Values equal to or greater than 0.5 for both sexes were considered abdominal obesity [36].

### 2.3. Blood Pressure

Systolic and diastolic blood pressure (SBP and DBP) were measured by the auscultatory method, using a pediatric stethoscope and aneroid sphygmomanometer (Premium©, Medical Instruments, Wenzhou, China), previously calibrated, with an adequate pediatric cuff size (10 to 35 cm). All the measures were performed following national [37] and international guidelines [38]. Three consecutive measurements were taken with an interval of two minutes between them, on three different days. The SBP was defined by the first Korotkoff sound (phase I), and the DBP by the disappearance of the Korotkoff sound (phase V) [37,38]. The mean of the average values obtained on the three days was considered. Participants were classified as pre-hypertensive when the mean SBP and/or DBP measurements were ≥90th percentile; and hypertensive, when the mean of the measurements was ≥95th percentile, according to age, sex, and height percentiles [37,38]. Both pre-hypertension and hypertension were considered as altered blood pressure.

### 2.4. Food Consumption Data

The food consumption was evaluated by three 24-h dietary recalls (R24h) carried out on 3 non-consecutive days (one of them on the weekend) [39], with an interval of no more than 4 weeks [25]. This evaluation was performed by an interview in which the children were asked and they reported what they had eaten in the previous 24 h [40]. The multiple-pass method was used to stimulate the child to remember the food ingested [41]. Food cited at least once by the children was included in the list. To facilitate the recording of the quantities consumed, an album of products and food developed by the researchers was used [42]. The conversion to grams and/or milliliters was performed according to the standardization of Pinheiro et al. [43]. The total energy value was estimated using the software ADS Nutri (Nutritional System, Rio Grande do Sul, Brazil, version 9.0). Foods that were not on the reference table were registered in the software by consulting the nutritional composition table of the foods consumed in Brazil [44] and the information on the labels [19].

In order to reduce the risk of inter-rater error, each participant was always assessed by the same person. The 142–146 personal variation in the food consumption was determined with 10% of the children evaluated, who were randomly selected. The adjustment of the distribution of energy intake was performed by removing the effect of intrapersonal variability, using the method proposed by Iowa State University [45].

The children reported 195 different items. These items were divided into three main groups, based on the four groups proposed by the Food Guide for the Brazilian Population (2014) [46], which was based on the NOVA international classification [15]. Group 1 (G1) was represented by unprocessed or minimally processed food, defined as those that were purchased for consumption without undergoing any change after leaving nature or were subjected to minimal changes, respectively (e.g., egg, milk, fruits, vegetables, fresh meat, etc.). Culinary preparations based on one or more unprocessed or minimally processed foods were also included in this category. These preparations include the food used as the main component of a recipe and all other ingredients, such as salt, sugar, vinegar, and oils [14]; Group 2 (G2) was composed of culinary ingredients, obtained from unprocessed foods or directly from nature (e.g., butter and sugar) and processed food, corresponding to products manufactured from the addition of some culinary ingredient to unprocessed or minimally processed foods (e.g., bread and cheese). Finally, Group 3 (G3) included ultra-processed food, which are formulations manufactured using series of processes and several ingredients, many exclusively for industrial use, and typically including little or no fresh food (e.g., soda, snacks, ice cream, chocolates, and sausage) [15]. Based on this information, the total caloric intake (kcal/day) for each food group was estimated, as well as the percentage of caloric contribution to the total caloric intake of each child.

### 2.5. Quality Control of Information

All the evaluations were performed in the school by a trained team, which included nutritionists, physical education professionals, and nutrition undergraduate students. Quality control of the information was carried out in 3 steps: (1) theoretical-practical training of the team, (2) re-tests in random samples of children, (3) measurement of reliability with intra-class correlation coefficient (R) for anthropometry, blood pressure, and food consumption. The intra-class correlation coefficients were: height = 0.996; waist = 0.995; SBP = 0.973; DBP = 0.959; total kcal = 0.847.

### 2.6. Statistical Analysis

The normality of the data was analyzed by the Kolmogorov–Smirnov test, as well as by inspection of histograms and evaluation of asymmetry. Numerical variables are described as mean and standard deviation or median and interquartile range (P25 and P75), while categorical variables are described as absolute and relative frequencies, with their respective 95% confidence intervals.

Linear regression analyses were performed to investigate the association between the percentage of calories of food, according to processing group (unprocessed or minimally processed; culinary ingredients and processed foods; ultra-processed foods) and anthropometric indicators and blood pressure. For all analyses, a crude univariate model and a multivariate model adjusted for age, sex, and total caloric intake were performed, with waist circumference included as a covariate when the association between food consumption and blood pressure was investigated.

For all models, collinearity, homoscedasticity, normality of residues, independence of residues, and linearity were verified. The data are expressed as regression coefficients (β), with their respective 95% confidence intervals, adjusted determination coefficient (r^2^) and significance values. All analyses were performed using the Statistical Package for the Social Sciences (SPSS, Inc. Chicago, IL, USA, version 25.0), with a significance level of *p* < 0.05.

## 3. Results

**Figure 1:**
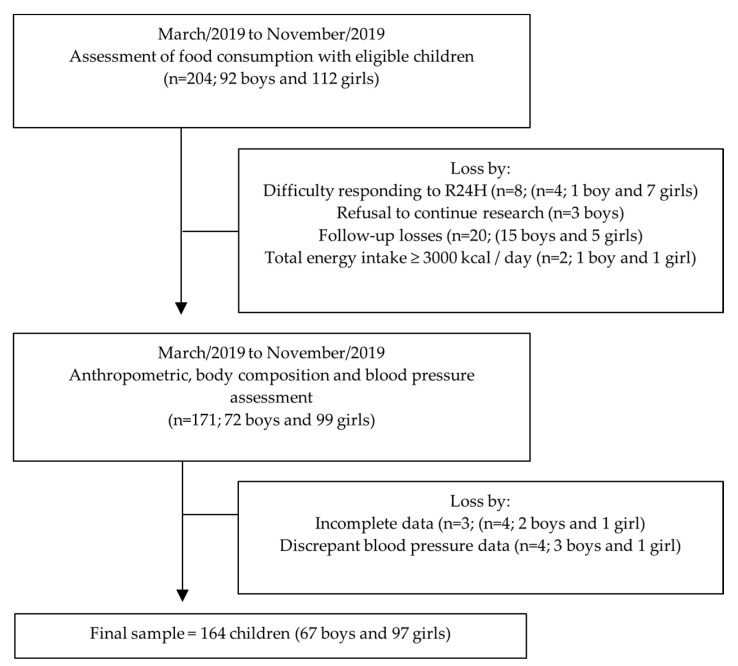
Flowchart of children included in the analyses.

Of the 204 potentially eligible schoolchildren, 164 children completed all measures and participated in the present study (Figure 1). As can be seen in Table 1, 40.8% (*n* = 80) of the children presented weight above the normal, being 20.1% overweight (OW) and 28.7% obese (OB). Abdominal obesity was present in 28.0% (*n* = 46) of children and altered blood pressure was observed in 10.9% (*n* = 18) of children.

The mean total energetic consumption was 1762.76 kcal/day (95% CI: 1705.28–1820.24 kcal/day). The percentages of the energy from each food group were 45.42% (95% CI: 43.42–47.42%) from G1, 10.88% (95% CI: 9.64–12.12%) from G2, and 43.70% (95% CI: 41.69–45.72%) from G3, representing more than one third of the total calories ingested by the children (Table 2).

Table 3 lists the food intake reported by the children. Bovine or pork meat and spaghetti were the major energy contributors in the unprocessed or minimally processed food group, corresponding to 7.30% and 6.34% of the total energy, respectively. Together, rice and beans contributed approximately 10% of the calories consumed daily. Among the group of culinary ingredients and processed food, the major caloric contribution was bread (6.92%). In the ultra-processed food group, sweet cookies and cakes (11.60%), followed by snacks and industrialized popcorn (4.96%), reconstituted meat products (4.42%), and sweets (4.15%) were the most commonly consumed.

There was no significant association between food consumption according to the degree of processing and the anthropometric indicators BMI, WC, and WHtR (Table 4). The caloric contribution of unprocessed or minimally processed foods was inversely associated with DBP, regardless of other adjustment variables; that is, for each 10% increase in the energy intake of unprocessed or minimally processed foods, there was a reduction of 0.96 mmHg in the DBP (β: −0.10; 95% CI: −0.19 to −0.01; r^2^ = 0.20) (Table 4). No association was observed between the caloric contribution of different food groups and SBP (*p* > 0.05).

## 4. Discussion

The main finding of the present study was that a 10% increase in energy consumption from unprocessed or minimally processed food was associated with a 0.96 mmHg decrease in DBP, even after adjusting for confounding factors. As far as we know, there is no evidence documenting the association between the degree of food processing and blood pressure in children. Although no association was found between the consumption of ultra-processed food and anthropometric indicators of obesity and blood pressure, high prevalences of overweight, abdominal obesity, and high blood pressure were observed in the children studied, along with a high consumption of ultra-processed foods.

The prevalence of overweight and obesity (20.1% and 28.7%, respectively) in this study was higher than the prevalence found in a previous study performed by Santos et al. [47], which showed a prevalence of 15.2% of overweight and 8.9% of obesity in 501 children between 7 and 10 years from the same municipality. This prevalence was also higher than in other countries, such as Canada (4–6 years old, OW 15.7%; OB 4.2%; severe obesity 4.2%), Poland (4–6 years old, OW 8.7% OB 3.3%), Hungary (6–8 years old, OW 14.2%; OB 12.7%), China (6–7 years old, OW + OB = 19.7%), Portugal (6–10 years old, OW15.9%; OB6.1%) [48,49,50,51,52]. Moreover, a great study involving 3.5–5.5-year-old children from six European countries also found a smaller prevalence (Belgium OW 9.4% OB 2.0%; Bulgaria OW 11.5%, OB 3.7; Germany OW 8.6%, OB 1.4%; Greece OW 14.9%, OB 5.7; Poland OW 10.4%, OB 2.3%; Spain OW 12.0%, OB 2.8%; overall OW + OB = 14.5%) [53].

However, a study performed in Spain with 5–11-year-old children have found prevalence of 25.8% for overweight and 13.2% for obesity [54] and another one performed with 6–11-year-old children in the USA have found a prevalence of 18.4% for obesity and 5.2% for severe obesity [55]. In Australia, the prevalence of overweight was 20.6%, for obesity was 4.9, and for severe obesity was 1.9% in 7–15 years old children [56], and in Italy, Barba et al. [57] observed a prevalence of 25.2–26.7% for overweight and 20.1–21.1% for obesity in children aged 6–11.

Various factors may explain the differences found among countries, such as the consumption of ultra-processed foods or of specific ultra-processed foods (soft drinks/sweetened beverages) [27,58], overweight/obese parents [53] and advances in policies to promote healthy eating, especially with regard to the marketing of food for children [59]. Socioeconomic factors may also influence the prevalence of OW/OB, as children from families with low family income or socioeconomic status are more likely to be overweight/obese [48,53]. Moreover, Batalha et al. (2017) [60] showed that low maternal education was associated with the high consumption of processed and ultra-processed foods by children, which may be associated with a high prevalence of obesity [20,22].

The prevalence of altered blood pressure was lower than that observed in a cross-sectional study conducted with 373 Cuban children aged 8 to 11 (hypertension: 5.1%; pre-hypertension: 32.2%) [61]. In Italy, 9.9% of the boys and 13.9% of the girls presented pediatric hypertension [57]. However, the percentage of hypertensive children in the present study follows that observed in Brazil. De Souza et al. (2017) [4], showed that 2.1% of children aged 7 to 10 years in public schools of Vitória, Espírito Santo were hypertensive. Moreover, hypertension was reported in 5% of boys and in 3% of girls from a Iranian study with 6–18 years old children [62]. It was reported that individuals aged 38 years at a high risk of developing hypertension and cardiovascular comorbidities (e.g., dyslipidemia) had high blood pressure when they were 7 years old, and that a higher body mass index resulted in higher levels of blood pressure [8]. It is known that overweight and obese children have higher blood pressure and more risk factors for cardiovascular diseases, compared to those with normal weight, due to factors such as imbalance in the pro and anti-inflammatory activities of adipocytes, endothelial dysfunction, and early atherosclerosis [2,57,63,64,65]. These results reinforce the importance of regular assessments of blood pressure and its risk factors in school-age children.

The consumption of unprocessed or minimally processed foods was similar to the one found in children (2–10 years old) in southern Brazil (47%) [19]. In the present study, contrary to what was observed by De Melo et al. [24], no association was found between the consumption of unprocessed or minimally processed foods and anthropometric indicators of obesity. On the other hand, an inverse association was observed between consumption of unprocessed or minimally processed foods and DBP. A study with 606 Portuguese adolescents showed that, in girls, an increase in 100 g of fruit per day was significantly associated with a 0.5 mmHg decrease in DBP [β: −0.005 mmHg (95%CI: −0.01; −0.0002) (*p* = 0.038)], after adjustment for confounders [66]. Gilardini et al. (2015) [67] showed that obese and hypertensive children and adolescents consumed less vegetable protein than normotensive children and adolescents (6.5 ± 1.6 vs. 7.1 ± 1.5%; *p* < 0.05). The authors also observed that BP was negatively related to vegetable protein (systolic r = −0.120; *p* < 0.05; diastolic r = −0.267; *p* < 0.01).

It is not a consensus whether systolic or diastolic blood pressure should be chosen as the diagnostic criterion to be used to define the presence of SAH. What is becoming apparent is that SBP and DBP identify distinct hypertension phenotypes, with the latter being related to an increase in peripheral vascular resistance and mean arterial pressure (or type 1 hypertension) [68]. In adolescents, DBP is a more powerful predictor of cardiovascular disease in adulthood than SBP [69]. Considering the importance of earlier detection and prevention of risk, the authors emphasize the risk associated with high diastolic blood pressure in this population. As far as we know, there are no studies showing this association in children, but it is reasonable to think that assessing DBP in children would be useful for the prevention of cardiovascular diseases.

A diet that includes unprocessed or minimally processed foods is beneficial for cardiometabolic health [70]. One factor in food processing that may play a role in this association is the nutritional composition of the final product. Unlike ultra-processed foods, unprocessed or minimally processed foods have low energy and sodium density and are rich in fibers and protein [14]. A study with adolescents showed that the increase in fiber intake to daily recommendations was associated with expected reductions in diastolic blood pressure of 5.2 and 3.0 mmHg in boys and girls, respectively, after statistical adjustments [71]. Dietary fiber is a non-digestible form of carbohydrates that is present in foods such as fruits, vegetables, oats, whole grains, and seeds. Its effect in reducing blood pressure is secondary to the control of risk factors for hypertension. Due to the physicochemical properties, fibers can contribute to a reduction in the risk of weight gain and central obesity; improvement in the response to insulin, by promoting slow gastric emptying, and improvement in vascular and endothelial function, by favoring the reduction in cholesterol levels and reduction in systemic inflammation [72]. A systematic review and meta-analysis of 85 studies including 58,531 children and adolescents showed that sodium intake was associated with systolic and diastolic blood pressure and that an average reduction in sodium intake of 1.2 g/day (range: 0.2–4.3 g/day) reduced diastolic blood pressure by 1.2 mm Hg (95% CI: 0.4, 1.9). The authors reinforce the importance of limiting sodium consumption in childhood, which can be achieved by including unprocessed or minimally processed foods in the diet [73].

The caloric contribution of ultra-processed foods was high and corroborates the findings by Costa et al. [27], who reported an average contribution of 47.8 ± 8.9% (753.8 ± 191.0 kcal/day) in children aged 8, in the city of São Leopoldo, Rio Grande do Sul, Brazil. The inclusion of ultra-processed foods in a child’s diet can be influenced by several factors, such as maternal education [60], the child’s age [19], and advertisements [74]. In the present study, the availability and access to these foods in and around the evaluated schools may also have favored the consumption of these foods. To combat this scenario, national policies are necessary to promote healthy eating practices for children, including the prohibition of marketing and advertising of ultra-processed foods in school spaces. However, to date, there is only one official document that establishes guidelines for the Promotion of Healthy Eating in public and private schools in Brazil [75]. The Food Guide for the Brazilian population recommends avoiding the consumption of ultra-processed foods and making unprocessed or minimally processed foods the basis of the diet [46]. Therefore, assessing the consumption of ultra-processed foods during childhood is important because eating habits acquired during this period, whether healthy or not, tend to remain in adulthood [76].

The consumption of ultra-processed food was not associated with the anthropometric indicators of obesity and blood pressure, corroborating a previous study [24]. However, longitudinal studies conducted with Brazilian children reinforce the possible negative effects of ultra-processed foods on children’s health, such as increased total cholesterol and low-density lipoprotein (LDL) [25], triglycerides [26], and waist circumference [27]. These changes are associated with an increased risk for cardiometabolic disease [5,77]. Another consequence of the consumption of ultra-processed foods concerns the negative impact on the nutritional profile of food [13,14]. In addition, highly processed foods represent a significant source of advanced glycation end products (AGEs), mainly due to high exposure to heat. AGEs are heterogeneous compounds derived from non-enzymatic glycation reactions from interactions between reducing sugars or oxidized lipids and proteins or nucleic acids. The pathological effects of AGEs are related to their ability to alter the chemical and functional properties of proteins and activate intracellular pathways promoting oxidative stress [78,79]. Studies indicate the possible effect of dietary AGEs on inflammation and cardiometabolic dysfunction in adults and in the pediatric population [80,81,82].

It is likely that the absence of an association in the present study is due to the age of the children, who may not have consumed these types of foods for long enough to induce metabolic and/or cardiovascular alterations, which may appear in the following phases of life. Moreover, it should also be noted that the etiology of obesity and hypertension is multifactorial, involving factors such as genetics, physical inactivity, family lifestyle, and psychological factors, which were not assessed in the present study [11,77].

The present study has limitations: (i) the cross-sectional design prevents the attribution of causality between the variables; (ii) the lack of socioeconomic information and evaluation of the level of physical activity did not allow an investigation of the influence of these factors on the associations; and (iii) the small sample size implies caution in the generalization of the results, although the sample was statistically sufficient to detect the demonstrated relationships; and (iv) the lack of information on the amount of calories for each main nutrient (e.g., carbohydrates, proteins, lipids). However, we provided details on the amount of calories for each specific food shown, although we encourage further research to assess the association of each specific nutrient with anthropometric and blood pressure indicators. Furthermore, the fact that the children completed the R24H may have led to inconsistencies influenced by recall bias. However, to minimize this, the 355–357 pass method was used, evaluations were carried out individually, the interviewers were well trained, and quality control was carried out. On the other hand, there are many strengths: this is the first study to analyze the impact of food consumption according to the degree of processing on the anthropometric indicators of obesity and blood pressure in a sample of children from the interior of PE. In addition, the use of 3 R24h allowed the collection of a greater diversity of information on the food consumed.

## 5. Conclusions

The consumption of unprocessed or minimally processed foods was inversely associated with diastolic blood pressure. In addition, we observed that the caloric contribution of ultra-processed foods was high in the children’s diet. These results reinforce the need to create and implement preventive strategies for food and nutrition education in the school environment, among which the increase in consumption of unprocessed or minimally processed foods should be emphasized, aiming at the prevention and treatment of hypertension in children.

## Figures and Tables

**Table 1:** General characteristics of children aged 7–10 years. Vitória de Santo Antão, Pernambuco, Brazil (*n* = 164).

Variables	*n* (%)	95% CI
Sex		
Boys	67 (40.9)	33.5–48.5
Girls	97 (59.1)	51.5–66.5
Nutritional status		
Underweight	1 (0.6)	0.0–2.7
Normal Weight	83 (50.6)	43.0–58.2
Overweight	33 (20.1)	14.5–26.7
Obese	47 (28.7)	22.1–35.9
Abdominal obesity		
Waist circumference (>90th percentile)	46 (28.0)	21.5–35.2
Waist-height ratio (>0.5)	58 (35.4)	28.3–42.9
Blood pressure		
Normal	146 (89.0)	83.6–93.2
Pre-hypertension	13 (7.9)	4.4–12.7
Hypertension	5 (3.0)	1.1–6.4

CI: Confidence interval.

**Table 2:** Description of total energy intake and energy percentages from each food group according to the degree of processing among children aged 7–10 years old. Vitória de Santo Antão, Pernambuco, Brazil (*n* = 164).

Variables	Mean ± SD	95% CI
Caloric intake (kcal/day)		
Total	1762.76 ± 372.79	1705.28–1820.24
Unprocessed or minimally processed foods ^†^	790.19 ± 246.40	752.20–828.18
Culinary ingredients and processed foods	194.10 ± 148.28	171.24–216.96
Ultra-processed foods	778.47 ± 308.83	730.85–826.09
Contribution of daily intake (% of total energy intake)		
Unprocessed or minimally processed foods ^†^	45.42 ± 12.96	43.42–47.42
Culinary ingredients and processed foods	10.88 ± 8.07	9.64–12.12
Ultra-processed foods	43.70 ± 13.06	41.69–45.72

SD: standard deviation. CI: Confidence interval. ^†^ Includes culinary preparations based on these foods.

**Table 3:** Average absolute daily caloric intake and energy percentages of consumption of unprocessed or minimally processed foods, culinary ingredients and processed foods, and ultra-processed foods by children aged 7–10 years. Vitória de Santo Antão, Pernambuco, Brazil (*n* = 164).

Food Groups and Subgroups	Absolute Ingestion (kcal/day)	Relative Intake (% Total kcal)
**Unprocessed or minimally processed foods ^†^**	**790.19**	**45.42**
Beef	129.68	7.30
Spaghetti ^a^	108.31	6.34
Rice	95.65	5.53
Beans	75.57	4.48
White meat	66.63	3.91
Corn, munguzá, couscous, bread	66.10	3.79
Fruit juice	44.49	2.58
Milk	41.78	2.36
Eggs	40.53	2.27
Fruits	26.18	1.52
Cassava flour, tapioca and mush meal	21.66	1.24
Homemade popcorn	17.05	0.93
Roots and tubers	16.12	0.86
Coffee and tea	8.85	0.53
Homemade cake (manioc, corn, wheat, and orange)	7.41	0.40
Homemade soup	4.84	0.31
Vegetables and legumes	3.41	0.21
Fish and other seafood ^b^	2.16	0.11
Other foods and culinary preparations ^c^	13.76	0.75
**Culinary ingredients and processed foods**	**194.10**	**10.88**
Bread	123.09	6.92
Cheese	24.00	1.29
Sugar	20.40	1.18
Processed meat	8.23	0.45
Butter	7.55	0.41
Canned sardines	6.04	0.33
Other processed foods ^d^	4.78	0.29
**Ultra-processed foods**	**778.47**	**43.70**
Sweet cookies and cakes	203.40	11.60
Industrialized popcorn and snacks	87.41	4.96
Reconstituted meat products ^e^	80.21	4.42
Sweets and candies ^f^	75.28	4.15
Soft drinks and industrialized fruit juices	69.19	3.95
Salty crackers	64.32	3.51
Fast food snacks ^g^	58.54	3.29
Sweetened milk drinks	36.22	2.15
Lasagna and savory pies	19.71	1.03
Instant noodles	17.20	0.86
Loaf of bread, hamburger and similar	16.04	0.92
Packet roasted peanuts	11.63	0.65
Chocolate powder	9.04	0.52
Mix of flavored and sweetened corn starch	6.56	0.41
Industrialized flour	6.42	0.33
Margarine	5.75	0.34
Industrialized sauces ^h^	5.22	0.27
Other ultra-processed foods ^i^	6.31	0.34
Total	1762.76	100.00

^†^ Includes culinary preparations based on these foods. ^a^ Includes pasta. ^b^ Includes crab and shrimp. ^c^ Pancake, mashed potatoes, coconut water, rolled oats, cashews, sugar cane juice and molasses and avocado smoothie. ^d^ Canned guava and banana, paçoca, corn and pea sweets. ^e^ hamburger, sausage, pepperoni, bologna, sausage, nuggets. ^f^ Gum, lollipop, candies, lozenge, chocolates, puddings and ice cream. ^g^ Hamburger, hot dog, pizza and fried and baked snacks. ^h^ Mayonnaise, Mustard and Ketchup. ^i^ Creamy curd, breakfast cereals and jams.

**Table 4:** Linear regression coefficient for the association between the caloric contribution of daily intake of unprocessed or minimally processed foods, culinary ingredients and processed foods, and ultra-processed foods with anthropometric indicators of obesity and systolic and diastolic blood pressure among children aged 7–10 years old. Vitória de Santo Antão, Pernambuco, Brazil (*n* = 164).

	BMI (kg/m^2^) ^a^			WC (cm) ^a^			WHtR ^a^			SBP (mmHg) ^b^			DBP (mmHg) ^b^		
	β	IC (95%)	R^2^	β	IC (95%)	R^2^	β	IC (95%)	R^2^	β	IC (95%)	R^2^	β	IC (95%)	R^2^
Unprocessed or minimally processed foods	−0.006	−0.058 to 0.047	0.050	0.005	−0.126 to 0.136	0.071	0.001	−0.001 to 0.001	0.027	−0.021	−0.116 to 0.073	0.199	−0.096	−0.186 to −0.006	0.202
Culinary ingredients and processed foods	0.025	−0.059 to 0.108	0.052	0.083	−0.123 to 0.288	0.075	0.001	−0.001 to 0.002	0.029	0.001	−0.148 to 0.151	0.198	0.044	−0.099 to 0.188	0.180
Ultra-processed foods	−0.004	−0.057 to 0.048	0.050	−0.037	−0.167 to 0.092	0.073	0.001	−0.001 to 0.001	0.029	0.020	−0.073 to 0.114	0.199	0.076	−0.013 to 0.166	0.194

WC, waist circumference. BMI, body mass index. WHtR, waist-to-height ratio. CI, Confidence interval. DBP, diastolic blood pressure; SBP, systolic blood pressure. ^a^ Adjusted by age, sex and total caloric intake (kcal/day). ^b^ Adjusted by age, sex, total caloric intake (kcal/day) and waist circumference.
